# Epidemiological survey of tularemia in Ilam Province, west of Iran

**DOI:** 10.1186/s12879-019-4121-1

**Published:** 2019-06-07

**Authors:** Saber Esmaeili, Ahmad Ghasemi, Razi Naserifar, Ali Jalilian, Leila Molaeipoor, Max Maurin, Ehsan Mostafavi

**Affiliations:** 1 Department of Epidemiology and Biostatistics, Research Centre for Emerging and Reemerging infectious diseases, Pasteur Institute of Iran, No. 69, Pasteur Ave, Tehran, 1316943551 Iran; 2 National Reference laboratory for diagnosis and research on Plague, Tularemia and Q fever, Research Centre for Emerging and Reemerging infectious diseases, Pasteur Institute of Iran, Akanlu, Kabudar Ahang, Hamadan, Iran; 3 Department of Bacteriology, Faculty of Medical Sciences, Tarbiat Modares University, Tehran, Iran; 4 Department of Parasitology, Faculty of Health, Ilam University of Medical Sciences, Ilam, Iran; 5 Department of Epidemiology, School of Public Health, Iran University of Medical Sciences, Tehran, Iran; 6 Student Research Committee, Faculty of Public Health Branch, Iran University of Medical Sciences, Tehran, Iran; 7 Centre National de Référence Francisella tularensis, Laboratoire de Bactériologie, Institut de Biologie et de Pathologie, CHU Grenoble Alpes, Grenoble, France; 8 TIMC-IMAG, CNRS/UGA, UMR5525, Université Grenoble Alpes, Grenoble, France

**Keywords:** Tularemia, *Francisella tularensis*, Seroprevalence, Iran, High-risk population

## Abstract

**Background:**

*Francisella tularensis* is the causative agent of tularemia in humans and a large number of animal species. Considering recent evidence of the circulation of this bacterium in different parts of Iran, especially in the western provinces, the aim of current study was to determine the tularemia seroprevalence in the human population living in Ilam Province.

**Methods:**

In 2015, 360 serum samples were collected from five groups of people: ranchers (*n* = 112), farmers (*n* = 79), butchers and slaughterhouse workers (*n* = 61), Nature Conservation Officers (*n* = 34), and referents of medical diagnostic laboratories (*n* = 74). These samples were tested for the presence of anti- *F. tularensis* IgG antibodies using the ELISA method.

**Results:**

According to the ELISA manufacturer cutoffs, we found that 10 (2.78%) and 9 (2.5%) sera, respectively, were positive or borderline for *F. tularensis* IgG antibodies. The highest tularemia seroprevalence was observed among farmers (7.59%).

**Conclusions:**

Our results strongly support the circulation of tularemia in Ilam Province. Because no human tularemia case has been reported so far in this province, we recommend specific education programs to increase knowledge of local health care professionals about this important zoonotic disease.

## Background

*Francisella tularensis* is a Gram-negative, intracellular bacterium, causing tularemia in humans and many animal species [1]. This pathogen is considered a biological threat agent because it can induce severe diseases even at a low infectious dose, it can be spread by aerosols, it can survive for months in water and soil environments, only a limited number of antibiotics can be used for treatment, and no vaccine is available for prevention of tularemia [2]. The species *F. tularensis* classically includes four subspecies: *tularensis* (type A), *holarctica* (type B), *mediasiatica* and *novicida*. Only the two former subspecies are considered causative agents of tularemia in the world. Type A strains are mostly found in North America. Type B strains are spread throughout the northern hemisphere, and thus is the only subspecies causing tularemia in Europe and Asia [3]. Type B strains have been recently detected in Australia [4].

*F. tularensis* can infect many vertebrate and invertebrate animals. Small rodents and lagomorphs (rabbits and hares) are considered the primary source of human infections. Domestic animals (especially lambs and cats in the United States) can occasionally be infected with (or carriers of) *F. tularensis*, and may transmit the disease to humans [2]. At-risk groups for tularemia include laboratory staff, farmers, ranchers, hunters, veterinarians, Nature Conservation Officers, butchers and slaughterhouse workers [5]. The routes of tularemia transmission to humans are numerous: direct contact with infected animals (especially hares); consumption of *F. tularensis*-contaminated meat or water; arthropod bites (mainly ticks, and mosquitoes in specific areas); and exposure to a contaminated water or soil environment [1, 3, 6–8].

The clinical manifestations of tularemia in humans vary from an asymptomatic infection to very severe diseases, which may lead to death [9]. After a mean incubation period of 3–5 days, the disease begins often with flu-like symptoms such as fever, chills, weakness, myalgia, joint pain, sore throats and headache. Then, depending on the route of infection, the disease may evolve to one of six main clinical forms: glandular, ulceroglandular, oropharyngeal, oculoglandular, pneumonic, and typhoidal [3]. Type A infections are usually more severe than type B, and the mortality rates in untreated patients are 10–40 and 1%, respectively [9].

In Iran, the first human tularemia case was reported in 1981 from the western part of the country (Kurdistan province). The patient suffered from a glandular form of tularemia, with fever, chills, myalgia, fatigue, headache, anorexia and severe enlargement of the inguinal lymph nodes [10]. Since then, no other tularemia case has been reported in Iran [5]. Recent seroepidemiological studies, however, have reported significant prevalence of anti-*F. tularensis* antibodies in the Iranian population [11–13]. In a recent study, *F. tularensis* has been detected in rabbits and rodents in Iran by molecular methods [14]. On the other hand, Turkey (a neighboring country in northwestern Iran), reported annually 400–2000 human tularemia cases during the 2009–2012 period [8]. In fact, very few recent studies have evaluated the status and epidemiology of tularemia in Iran. The aim of the current study was to investigate the tularemia seroprevalence among high-risk groups of individuals in the western province of Ilam.

## Methods

### Study area

**Fig. 1:**
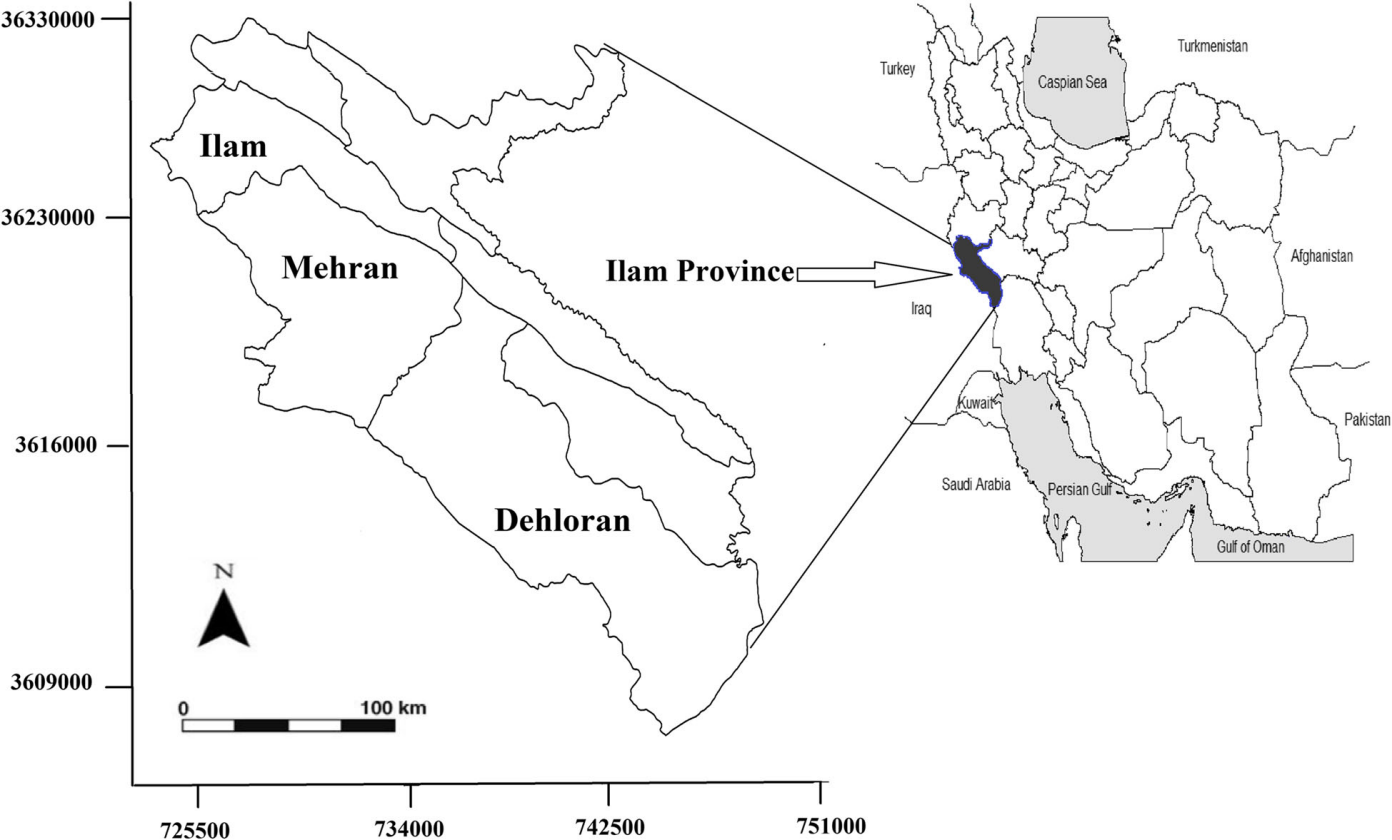
Sampling site of Ilam Province in West of Iran to study tularemia infection, 2015

This study was conducted in Ilam Province, western Iran, in 2015 (Fig. 1). The province population is approximately 600,000, and it is located in a mountainous region with a Mediterranean climate. This province covers an area of 20,000 km^2^, about 1.2% of Iran.

### Sampling

Blood samples were collected in three counties located on the western part of Ilam Province: Ilam, Dehloran and Mehran (Table 1). Patients included in the study were over 18 years old and belonged to one of four at-risk populations: ranchers, farmers, butchers and slaughterhouse workers, and Nature Conservation Officers. Referents of medical diagnostic laboratories (healthy people without any clinical symptoms) from the same area were selected as the control group. Informed consent was obtained from all selected individuals. For each participant, a 5 ml blood sample was taken and all samples transferred immediately to the laboratory for serum extraction. Serum samples were then stored at − 20 °C. All sera were sent to the National Reference Laboratory for Plague, Tularemia and Q fever in the Pasteur Institute of Iran for serological investigations.

**Table 1:** Population of the sampling sites of the studied areas in Ilam Province, western Ilam

		Area of residence			
County	Population	Urban	Rural	Nomads	No. of samples
Ilam	235,144	199,861	35,230	53	141
Dehloran	65,630	41,183	24,396	51	100
Mehran	29,797	19,186	10,516	95	118

Demographic and epidemiological data from people included in the study were collected using two questionnaires. The demographic questionnaire included the following information: age, sex, education, occupation, marital status, county and place of residence. The second questionnaire collected risk factors for *F. tularensis* exposure: owning animals; hunting; consumption of meat from wild animals; contact with a sick animal or its corpse; bites from arthropods (ticks, fleas and mosquitoes); splashing blood or other body fluids on the face or body; consumption of non-pasteurized milk or dairy products; cutting hands or other parts of the body during work with animals; use of personal protective equipment; and in general knowledge of zoonosis and protective measures against this infectious risk.

### Serology

All sera were tested for the presence of *F. tularensis* IgG antibodies using the commercial ELISA kit (Serion ELISA classic *Francisella tularensis*, Virion/Serion GmbH, Würzburg, Germany) according to the manufacturer’s instructions. The retrieved ODs were evaluated according to the protocols of Serion/Verion and the IgG was reported in a quantitative way. The titer of IgG antibody was calculated using a logistic- log model calculation in U/ml. Using cut-off titers advocated by the manufacturer serological titers were reported as positive (antibody titer > 15 U/ml), borderline (10–15 U/ml), or negative (< 10 U/ml).

### Statistical analysis

The data was analyzed using Stata software version 11 (StataCorp, College Station, TX, USA). The potential influence of tularemia risk factors on the observed seroprevalence rates was investigated by logistic regression analysis. *P*-values less than 0.05 were considered statistically significant.

## Results

We collected 360 serum samples from the five studied groups, including ranchers (*n* = 112), farmers (*n* = 79), butchers and slaughterhouse workers (*n* = 61), Nature Conservation Officers (*n* = 34), and referents of medical diagnostic laboratories (*n* = 74). Among these participants, 76.29% were males, 89.86% were married, and 45.18, 44.88 and 10.44% were living in urban, rural or nomadic areas, respectively. The mean (± SD) age of the tested subjects was 40.54 (±13.55) years old (range 18 to 78 years).

Of the 367 sera samples, 10 (2.78%) and 9 (2.5%) of participants were considered positive or borderline for anti-*F. tularensis* IgG antibodies, respectively. No present or past history of tularemia could be recorded in seropositive individuals. The highest and the lowest tularemia seroprevalence were observed in farmers (7.59%) and Nature Conservation Officers (0%), respectively (Table 2). Seroprevalence was almost the same in all the studied counties and in both genders. The age 31–40 years and over 50 years old had the highest and lowest seroprevalence, respectively. None of the investigated risk factors had a statistically significant relationship with tularemia seroposivity (Table 3).

**Table 2:** Results from the bivariate logistic regression analysis of demographic predictors of tularemia using unadjusted odds ratios (ORs)

Variable	Category	Number (% Positive Test)	Crude OR (95% CI)	P-Value
Occupation	Referents of the medical diagnostic labs	74(1.35)	1.00	
	Rancher	112(0.89)	0.67 (0.02–26.34)	0.802
	Butcher	61(3.28)	2.49 (0.19–74.92)	0.510
	Nature Conservation Officers	34(0.00)	0.0 (0.00–41.91)	0.688
	Farmer	79(7.59)	6.02 (0.86–142.6)	0.075
Gender	Male	275(2.91)	1.00	
	Female	85(2.35)	0.80(0.11–3.56)	0.84
Age (Year)	18–30	101(1.98)	1.00	
	31–40	97(5.15)	2.68(0.51–20.35)	0.259
	41–50	80(2.50)	1.27(0.13–12.4)	0.826
	> 50	81(1.23)	0.63(0.02–8.38)	0.759
Area of residence	Urban	158(2.53)	1.00	
	Rural	159(3.77)	1.51(0.41–6.16)	0.549
	Nomads	39(0.00)	0.0(0.0–4.55)	0.411
Education	Illiterate	81(2.47)	1.00	
	Primary	76(6.58)	2.76(0.53–21.13)	0.245
	Secondary	46(4.35)	1.79(0.18–17.66)	0.594
	Diploma	92(1.09)	0.44(0.01–5.82)	0.553
	Academic	63(0.00)	0.25(0.01–5.31)	0.314
Marriage	Single	36(5.56)	1.00	
	Married	324(2.47)	2.31 (0.32–10.5)	0.331
County	Dehloran	100(3.00)	1.00	
	Mehran	118(2.54)	0.85(0.14–5.03)	0.849
	Ilam	141(2.84)	0.95(0.19–5.17)	0.933

**Table 3:** Relationship between behavioral characteristics and tularemia seropositivity in Ilam Province, 2015

Variable	Number having the variable (%Seropositive)	Number not having the Variable(% Seropositive)	*P*-value
Attitude^a^	245(2.86)	113(2.65)	0.885
Splashing animal fluids on face/body	202(2.97)	99(3.03)	0.951
Exposure to ill or dying animals	86(0.00)	274(3.65)	0.063
Hunting/consumption of wild animal meat	228(3.95)	130(0.77)	0.148
Rabbit	8(0.00)	115(0.87)	0.935
Partridge	94(1.06)	29(0.00)	0.764
Fox	1(0.00)	124(0.81)	0.992
Mongoose	2(0.00)	121(0.83)	0.984
Emigrant/feral Birds	44(0.00)	79(1.27)	0.642
Mountain animals	88(1.14)	35(0.00)	0.715
Others**	3(0.00)	121(0.83)	0.976
Keeping animals	217(2.76)	139(2.88)	0.937
Cattle	77(3.90)	143(2.10)	0.466
Goats and sheep	197(2.54)	23(4.35)	0.610
Dogs and cats	100(2.00)	119(2.52)	0.832
Rabbit	8(0.00)	212(2.83)	0.799
Horse / Donkey	60(3.33)	160(3.12)	0.522
Hunting Meat Consumption (5 times or more)	21(0.00)	98(2.02)	0.677
Unpasteurized milk and dairy	179(2.23)	179(3.35)	0.544
Mosquito bites	247(2.83)	107(2.80)	0.865
Tick bites	32(0.00)	322(3.11)	0.383
Flea bites	42(0.00)	311(3.22)	0.277
Using disinfection tools	74(1.35)	81(1.23)	0.955
Disinfection of hands/face	75(1.33)	80(1.25)	0.961
Length of employment (20 years or more)	129(2.33)	154(4.55)	0.339
Cutting hand/year (5 times or more)	17(5.88)	226(3.54)	0.621

^a^See themselves as high risk for Zoonosis diseases

## Discussion

Our study strongly supports the presence of tularemia in Ilam Province. The overall tularemia seroprevalence among different populations studied was 2.78%, although we found a seroprevalence of 7.59% in farmers, which are probably more exposed to the *F. tularensis* animal reservoir. A much higher seroprevalence (14.7%) was previously reported in Kurdistan Province, western Iran [12]. A seroprevalence of 6.5% was also found in butchers and slaughterhouse workers in the Sistan and Baluchestan province, southeast of Iran [13].

The prevalence of tularemia in different parts of the world is highly dependent on people culture and lifestyle, although ecological conditions have an irrefutable role [15–17]. In Iran, the highest prevalence of tularemia has been observed among hunters (18%), and contact with wildlife has been shown to be a significant risk factor [12]. In the present study, there was no statistically significant relationship between tularemia seroprevalence and hunting or eating meat from wildlife animals. A rarer practice of hunting in the province of Ilam compared to other provinces of Iran may be one of the reasons for lower exposure to *F. tularensis* and lower tularemia seroprevalence.

Living in rural areas is usually considered a major risk factor for tularemia because of more frequent contact with wildlife animals [18, 19]. However, we found only weak difference of seroprevalence between rural and urban people of the study (3.77% versus 2.53%, NS). Similar studies in Iranian Kurdistan [12] and Turkey [20] have also reported non-significant differences between rural and urban populations. This probably means that people living in urban areas keep frequent contact with the rural environment. As in other studies [12, 13, 18], gender was not associated with a significant difference in tularemia seroprevalence. More surprisingly, a higher age was not associated with a higher seroprevalence of tularemia in our present study, while previous ones have shown a higher seroprevalence in older people [12, 18]. A possible explanation could be that tularemia has been reintroduced recently in Ilam Province, and thus middle-aged people, most at risk of tularemia, have higher seroprevalence.

Tularemia prevalence also depends on the main reservoirs and modes of transmission of *F. tularensis* to humans and animals. Several studies in Turkey have shown that tularemia outbreaks have occurred in recent years because of the consumption of contaminated water. A water-borne tularemia infection usually concerns a large population, of all ages, and all year round. Water sources are usually contaminated by the wildlife reservoir, including small rodents that are carriers of or infected by *F. tularensis*. The role of migratory birds has also been taken into consideration, because their respiratory secretions and feces can cause water contamination [21, 22]. Tularemia outbreaks have never been reported in Iran, which is not in favor of extensive water reservoir of tularemia. The role of ticks in the transmission of tularemia should be further evaluated as this mode of transmission is highly prevalent in most tularemia endemic countries.

Finally, the absence of any statistically significant risk factors in our study may be related to the small number of seropositive participants (10 out of 367 studied individuals). Therefore, it is recommended that the number of participants be higher in similar studies in areas with low tularemia seroprevalence.

Although we found anti-*F. tularensis* antibodies in several serum samples, no clinical case of tularemia has been reported so far in Ilam Province. The Serion ELISA method for detection of IgG antibodies directed against *F. tularensis* antigen is considered highly sensitive and specific [23]. Thus, our results highly suggest that tularemia is underdiagnosed in Ilam Province. *F. tularensis* strains circulating in Iran should probably be of type B as in the rest of Asia [1]. Type B strains usually cause infections of mild to moderate severity that can remained undiagnosed [18, 24]. On the other hand, very low attention has been paid to tularemia in training programs for physicians in Iran, and laboratories capable of confirming tularemia cases have only existed for a few years in Iran. It may be assumed that some tularemia patients have been treated blindly with antibiotics [12]. Therefore, failure to report human tularemia cases in Ilam Province is partly justifiable and does not necessarily mean that the disease is absent.

According to the high prevalence of tularemia in the neighboring countries of Iran, including Azerbaijan [18], Armenia [25] and Turkey [26], and the high tularemia seroprevalence we found in the present and in previous studies, there is a high potential for human infections with *F. tularensis* occurring in different parts throughout the country. Therefore, tularemia should be considered as a differential diagnosis in patients suffering from infectious diseases that have compatible clinical manifestations. Continuous monitoring of the presence of this bacterium should be carried out in different parts throughout Iran. Furthermore, to identify the potential reservoirs of *F. tularensis*, studies should be conducted to isolate this bacterium from various sources such as water and wild rodents.

One of the limitations of this study was the lack of an appropriate occupational group representing the general population. Referents of medical diagnostic labs were selected as the control group because of the possibility of taking blood samples from them. However, they may not be a representative sample of the general population. Nonetheless, the non-significant difference in the tularemia seroprevalence observed in this group compared to the other occupational groups may indicate that risk of exposure to *F. tularensis* in Ilam Province is unrelated to professional activities. Also, although the ELISA method has a high sensitivity and specificity for detection of anti-*F. tularensis* antibodies and this test has been previously used for seroepidemiological studies, other methods such as western blot, culture and PCR tests are required to confirm tularemia diagnosis [27, 28].

## Conclusions

Based on results of the present study and previous literature [5, 29, 30], tularemia is probably endemic in Iran. Further studies are needed to confirm the occurrence of human tularemia cases and evaluate their true incidence in Iran. Physicians and other health care professionals must be further sensitized about this zoonotic risk to achieve this goal.

## Data Availability

All data generated or analyzed during this study are included in this manuscript. The datasets used and/or analyzed during the present research project are available from the corresponding author upon reasonable request.
